# Application of the Non-Destructive NIR Technique for the Evaluation of Strawberry Fruits Quality Parameters

**DOI:** 10.3390/foods9040441

**Published:** 2020-04-06

**Authors:** Manuela Mancini, Luca Mazzoni, Francesco Gagliardi, Francesca Balducci, Daniele Duca, Giuseppe Toscano, Bruno Mezzetti, Franco Capocasa

**Affiliations:** Agricultural, Food and Environmental Sciences, Università Politecnica delle Marche, via Brecce Bianche 10, 60131 Ancona, Italy; m.mancini@pm.univpm.it (M.M.); l.mazzoni@staff.univpm.it (L.M.); S1071951@studenti.univpm.it (F.G.); francesca.balducci@staff.univpm.it (F.B.); d.duca@staff.univpm.it (D.D.); g.toscano@staff.univpm.it (G.T.); b.mezzetti@staff.univpm.it (B.M.)

**Keywords:** PCA, PLS, NIR spectroscopy, color, firmness, soluble solids, titratable acidity, strawberry

## Abstract

The determination of strawberry fruit quality through the traditional destructive lab techniques has some limitations related to the amplitude of the samples, the timing and the applicability along all phases of the supply chain. The aim of this study was to determine the main qualitative characteristics through traditional lab destructive techniques and Near Infrared Spectroscopy (NIR) in fruits of five strawberry genotypes. Principal Component Analysis (PCA) was applied to search for spectral differences among all the collected samples. A Partial Least Squares regression (PLS) technique was computed in order to predict the quality parameters of interest. The PLS model for the soluble solids content prediction was the best performing—in fact, it is a robust and reliable model and the validation values suggested possibilities for its use in quality applications. A suitable PLS model is also obtained for the firmness prediction—the validation values tend to worsen slightly but can still be accepted in screening applications. NIR spectroscopy represents an important alternative to destructive techniques, using the infrared region of the electromagnetic spectrum to investigate in a non-destructive way the chemical–physical properties of the samples, finding remarkable applications in the agro-food market.

## 1. Introduction

Strawberries are increasingly in demand in the worldwide fruit and vegetable market, from ever more demanding consumers with regards to appearance, taste and nutritional quality.

In order to guarantee the highest quality in terms of taste, color and consistency, strawberries should be harvested at almost complete maturation, as they are non-climacteric fruit.

Nowadays, strawberry fruits for market purposes are sorted based on their external quality such as a bright red color, uniform distribution, size, shape and absence of surface defects, but it is well known that internal quality parameters, such as taste, sweetness and acidity, are fundamental in the definition of fruit quality and strongly influence appreciation by the final consumer [1]. The qualitative characteristics of fruits are currently determined by destructive analyses which, if on the one hand guarantee clear and reliable information, on the other have different limits in terms of the amplitude of the product that can be analyzed due to the time necessary for carrying out the analyses. In fact, in the case of destructive methodologies it is not possible to apply them to an entire batch of a product but only to a certain representative number of fruits. The destructive methods to measure these parameters have been described in many studies. Sweetness is widely determined by Soluble Solids Content (SSC) which is normally measured through manual or automatic refractometers and expressed as °Brix [2,3]. Acidity is usually measured through colorimetric titration assays [4] or with a more accurate automatic titrator, which expresses this parameter as percentage of citric acid [5]. Firmness is a complex parameter that could be influenced by many factors, including the fruit’s internal structure and its chemical composition [6]. The most common method for the firmness evaluation is the puncture with a penetrometer, and the fruit firmness is expressed as g or N [7,8].

Over the last few years, researchers have been increasingly looking for non-destructive and fast techniques, particularly those based on optical properties [9,10], that could allow evaluation in real-time and produce analyses on a wider range of samples, requiring minimal preparation procedures [11]. Different optical methodologies have been successfully applied in the last few years to evaluate quality parameters as soluble solids content, acidity and firmness [6,12,13]. Among these techniques, Near-Infrared Spectroscopy (NIR) might be an important alternative; it uses the infrared region of the electromagnetic spectrum for investigating the chemical and physical properties of the samples in a non-destructive way. In fact, the chemical bonds of the molecules (OH-, CH- and NH- groups) vibrate at different energy levels and in different ways on the basis of the molecular structure of the sample analyzed [14]. This technique is already applied in different sectors, e.g., food [15,16], energy [17,18], pharmaceutical [19] and industrial sectors [20]. 

The best results are obtained when NIR spectroscopy is coupled with chemometrics. These techniques, in fact, are able to relate NIR spectra to the chemical properties of the analyzed samples, developing prediction models [21,22]. 

To our knowledge, many studies were recently conducted using NIR spectroscopy for the evaluation of strawberry sensorial and nutritional quality. The main weakness of those studies is usually the limited amount of fruits sampled, which could decrease the accuracy of the developed prediction models [23]. Furthermore there are usually only a few different varieties tested (sometimes just one), focusing attention mainly on different sample treatments (e.g., different postharvest storage conditions, different cultural systems and different pesticides treatments), and taking into account few qualitative parameters at the same time [24,25,26]. This means that the obtained results could be very useful for future research considering the same treatments but did not give an idea on the relation between the extent of qualitative parameters and the genetic variability of strawberries.

In this context, the aim of this work is to determine, in five strawberry genotypes, the main qualitative characteristics such as soluble solids content, acidity, fruit firmness and color through traditional destructive methods and NIR spectroscopy, developing prediction models for each parameter considered. These models are based on the comparison between the results obtained through the traditional lab analysis of the analyzed parameters and those detected by NIR spectroscopy. The implementation of non-destructive phenotyping methodologies will allow monitoring the quality parameters, to assure fruit quality before selling and to develop prediction models that will accelerate the breeding process. The expected results could be of great interest to breeders for the implementation of future research programs using non-destructive methodologies.

## 2. Materials and Methods

### 2.1. Plant Material and Fruit Sampling 

The assessment of the qualitative parameters was carried out on five strawberry genotypes: four cultivars (Cristina, Romina, Sibilla and Silvia) and one advanced selection (AN11,32,55) of the strawberry breeding program developed by the Department of Agricultural, Food and Environmental Sciences, Università Politecnica delle Marche.

The cultivation of the analyzed strawberries took place in the 2017–2018 season at the Extension and Research Center in Agriculture “P. Rosati”, in Agugliano (AN, Italy, 43°32′N–13°22′E).

The cultivation technique was typical of the Marche region. It involved the use of “A” type cold-stored plants, planted in an open field in the third decade of July.

The adopted experimental design was of four completely randomized blocks, with plots of 8 plants per block for a total of 32 plants for each genotype; this experimental design allows to standardize the variations of the fruit parameters due to the location of the plots in the field.

Twenty fruits were sampled for each plot, making a total of 80 fruits per each genotype (the four cultivars and the advanced selection), and they were sent to the lab for analysis.

The fruits were handled with care, individually numbered and subjected one by one to the different analyses, in the following order:

- NIR spectroscopic analysis (non-destructive technique);
- color analysis;
- firmness analysis.

At this point, fruits were frozen separately and after few days, analyzed one by one for:

- soluble solids content;
- titratable acidity.

### 2.2. Fruit Color, Firmness, Soluble Solids Content and Titratable Acidity

The color of the fruit was analyzed using an automatic reflectance colorimeter (Konica-Minolta Chroma-Meters CR-400, Tokyo, Japan), making two measurements on the opposite sides of each fruit. This tool, once applied to the surface of the fruit, is able to quantify the color, instantly providing three values: L*, a* and b*. Chroma (C*) is the parameter took into account in this study together with L* and it is calculated through the following equation:

(1) $$\mathrm{Chroma}={\left({\left(a^{*}\right)}^{2}+{\left(b^{*}\right)}^{2}\right)}^{1/2}$$

The fruit firmness was measured using a digital penetrometer (Fruit Firmness Tester, Turoni, Forlì, Italy) equipped with a star-shaped tip of 6 mm diameter which performed two measurements on the opposite sides of each fruit. The measurement was always performed by the same operator to minimize the error. Data were expressed as g.

The measurement of the Soluble Solids content (SS) allowed us to identify the sugar concentration of each single fruit and was carried out using a digital refractometer with automatic temperature compensation (Palette PR101α, Atago, Tokio, Japan). To carry out this analysis, each single frozen fruit previously used for color and firmness analyses was left to thaw and was then squeezed and the juice was extracted. A few drops of this juice were placed on the surface of the refractometer loading slide and the value in °Brix was measured two times for each fruit.

Titratable acidity indicates the whole amount of free acids present in strawberry juice (mainly ascorbic, malic, succinic and citric acid) which contribute, together with soluble solids, to define the taste of the fruit. This parameter was measured for each single fruit with an automatic titrator (Hanna HI84532, Hanna Instruments, Rhode Island, USA) following the manufacturer protocol: 5 mL of the juice previously extracted from each fruit was added to 45 mL of distilled water, and this solution was titrated. Titratable acidity is expressed by the instrument as a value corresponding to an equivalent amount of citric acid percentage. Some data for the acidity analysis is missing because for some fruits, it was not possible to obtain enough juice to titrate.

One-way analysis of variance was used to test the differences among strawberry genotypes for each analyzed parameter. Statistically significant differences (*p* ≤ 0.05) were determined with Tukey’s test. Statistical processing was carried out using STATISTICA software (Statsoft, Tulsa, OK, USA).

### 2.3. NIR Spectroscopy

In this work, the analysis was performed with the Fourier Transform (FT) NIR spectrophotometer (FT-NIR mod. Nicolet iS10, thermo, Massachusetts, United States), equipped with an integrative sphere. The range of interest was that of the near infrared region, specifically from 10000 to 4000 cm^−1^. The analysis was carried out using a continuous flow of nitrogen which aims to minimize the moisture content inside the instrument during the analysis, thus reducing the variability of the spectral analysis. Each spectrum was recorded by averaging 32 scans and with a resolution of 8 cm^−1^, resulting in 1557 absorption values. A background spectrum was scanned every hour, in which the entire electromagnetic signal is reflected, so as to reduce the variability associated to the environment and not to the sample.

Each fruit was analyzed twice. The first measurement was performed on a point along the fruit equator, the fruit was then turned 180° for the successive measurement.

### 2.4. Multivariate Data Analysis

Chemometrics was used to extract the useful information for the prediction of the parameters of interest. Before models computation, the spectral data were pre-treated using scatter correction methods and first and second derivatives in order to remove undesired physical phenomena [27] and each sample was averaged across the replicates.

Principal Component Analysis (PCA) was used as a qualitative method to show the statistical variance of all the samples on a chemical basis, clustering them depending on the spectral similarity. Based on the PCA results and the good separation trend among three of the five genotypes, an additional PCA was carried out considering only a part of the dataset.

Partial Least Squares Regression (PLS) was used to predict the parameters of interest on the full dataset. The technique works by evaluating the relationship between independent spectral values (X) and non-spectral dependent values (Y), maximizing the covariance of their scores [28]. PLS models were validated using venetian blind-cross validation (10 segments). 

To evaluate the performance of the developed model and its ability to estimate unknown samples, different parameters have been taken into account. In detail, the parameters are *r*^2^ or coefficient of determination, Root Mean Square Error of Cross Validation (RMSECV) and bias. Range Error Ratio (RER) and Ratio of Performance to Deviation (RPD) have also been computed. RER values <6 indicate a poor model, values between 7 and 20 indicate a model suitable for screening application and values over 20 indicate an excellent model [29]. Instead, RPD with values of 2-3, 3-4 and > 4 indicate a discrete, good and excellent model, respectively [30]. For each model the number of Latent Variables (LVs) was chosen considering the RMSECV plot against the number of LVs searching for a local minimum. In addition, U vs T (scores of Y and scores of X) plot and the loading plot were also investigated. 

Considering the huge number of samples analyzed, the best cross validation-PLS models obtained were also validated with an external test set. The dataset was split using Kennard-Stone algorithm: 279 samples were used as a training set and 120 samples as a test set. 

All the data analyses were performed using Matlab (ver. 7.10.0, The MathWorks) and in-house functions based on existing algorithms.

## 3. Results and Discussion

### 3.1. Destructive Lab Analyses 

The following table (Table 1) shows the results of the analyses concerning the fruit color, the firmness, the soluble solids content and the titratable acidity. 

From the color analysis, it is possible to notice that the highest value of brightness (L*) and Chroma (C*) have been reached by the fruits of the breeding selection AN11,32,55 (39.4 and 51.5, respectively), while the lowest value of L* was detected for Romina cultivar (35.7) and the lower value of C* was measured in the fruits of selection Silvia (43.2) (Table 1). Thus, the Romina and Silvia cultivars present fruits with low gloss, tending to be darker, while fruits of the selection AN11,32,55 resulted in a brighter and lighter color. This result was expected, as this selection was chosen for its very bright color.

To represent the results of the fruit firmness, the average values of the resistance of the fruits to penetration were taken. The fruits with a higher firmness were those belonging to the selection AN11,32,55 (661 g), unlike the cultivar Cristina, which showed fruits with a lower consistency (276 g). The Romina, Sibilla and Silvia cultivars presented fruits with an intermediate firmness.

Soluble solids is considered the parameter for the evaluation of fruit sugar content inducing the sweetness perceived by the consumer. The highest SS level was reached by the fruits of the Romina cultivar with an average of 8.4 °Brix, while the lowest were from the fruits of the selection AN11,32,55, with an average of 6.0 °Brix. Sibilla, Cristina and Silvia reached an average value of 7.7, 7.1 and 7.5 °Brix, respectively.

Titratable acidity is an important parameter in the perception of strawberry taste by the consumer, as the perception of acidity is accepted by consumers of northern areas while consumers of Asiatic countries demand a totally sub-acid fruit. Values shown in Table 1 for the fruits of the five genotypes fall within an average range of acceptable acidity. The fruits of Silvia resulted in higher acidity (0.77%), while Sibilla fruits are less acidic with an average value of 0.55%. The fruits of Romina, Sibilla and AN11,32,55 genotypes showed intermediate values.

### 3.2. PCA

**Figure 1:**
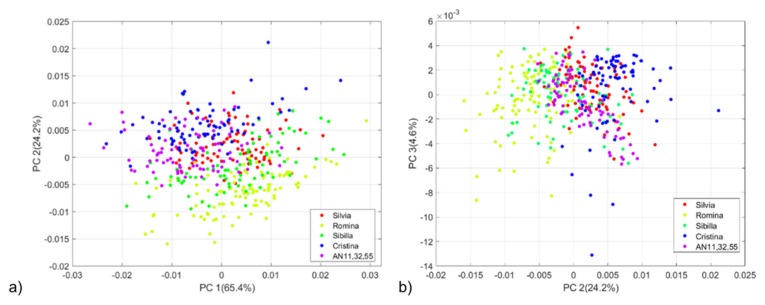
(**a**) Principal Component Analysis (PCA) score plot of the five strawberry genotypes (PC1 vs PC2); (**b**) PCA score plot of the five strawberry genotypes (PC2 vs PC3). Spectra were pre-treated with first derivative (Savitzky–Golay filter, 21 smoothing points, 2nd polynomial order). PC = principal component.

**Figure 2:**
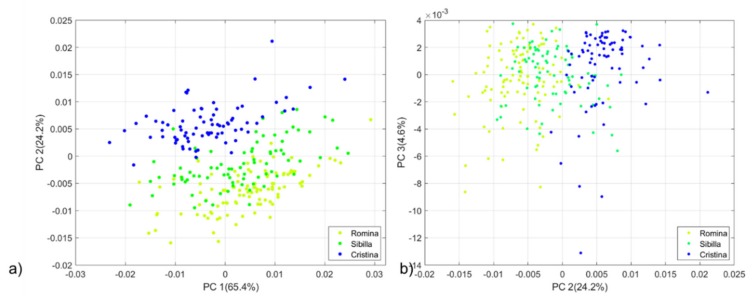
(**a**) PCA score plot of the Romina, Sibilla and Cristina cultivars (PC1 vs PC2). (**b**) PCA score plot of Romina, Sibilla and Cristina cultivars (PC2 vs PC3). Spectra were pre-treated with first derivative (Savitzky-Golay filter, 21 smoothing points, 2nd polynomial order). PC = principal component.

The PCA technique was used to explore the variability of our dataset and search for spectral differences among the five strawberry genotypes. The resulting dataset size consists of 800 observations (80 samples *x* 5 genotypes *x* 2 repetitions) *x* 1557 variables. Spectral data were pre-treated with first derivative (Savitzky-Golay filter, 21 smoothing points, 2nd polynomial order) before PCA computation. An outlier sample belonging to the Cristina cultivar has been detected from the PCA score plot and deleted from the successive data analysis. Accounting for 94.18% of the total initial variability, the three first principal components (PCs) were taken into account. The PCA score plots of all the possible combinations of PCs were investigated. In particular, the PC1 vs PC3 score plots did not show any separation among the genotypes (data not shown). Instead, PC1 vs PC2 and PC2 vs PC3 score plots showed a separation trend. Figure 1a,b show the PCA score plots of PC1 vs PC2 and PC2 vs PC3 respectively. Data from Silvia, Cristina and the AN11,32,55 selection are evenly distributed in the space, meaning that they produce fruits with similar characteristics. The similarity observed between Silvia and Cristina can be motivated by the fact that Silvia was originated in a breeding cross using Cristina as a female parent. Romina and Sibilla cultivars showed a more marked separation. Therefore, a new PCA was developed on a subset of the full dataset by considering Romina, Sibilla and Cristina cultivars. The new PCA score plots (Figure 2a,b) show a distribution trend along the PC2 where fruits of Cristina fits into the positive part while those for Romina and Sibilla fit into the negative part of the score plot.

**Figure 3:**
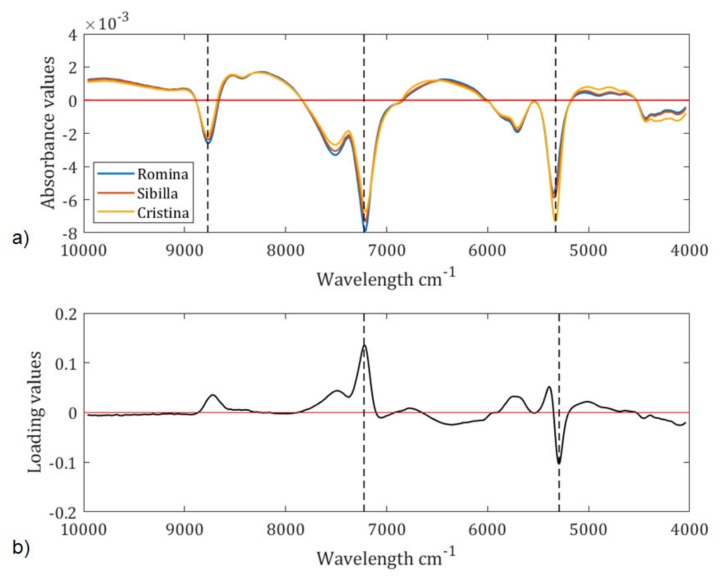
(**a**) Average spectra of Cristina, Sibilla and Romina cultivars pretreated using first derivative (Savitzky–Golay filter, 21 smoothing points, 2nd polynomial order); (**b**) Second loading. The dotted lines highlight the most important differences among the three cultivars.

To interpret the data and figure out which wavelengths are responsible for the sample grouping in the PCA score plot, the average spectra of the three cultivars and the second loading (i.e., the one that separates Cristina from Sibilla and Romina) were taken into consideration (Figure 3). Note that the interpretation of first derivative spectra is more complex than the raw spectra because a peak of maximum absorbance on the original spectra corresponds to zero in the 1st derivative [31]. For this reason, we have selected zero points corresponding to peak in the raw spectra for identifying the compounds more influent for the sample separation in the PCA score plot. 

Looking at the average spectra plot (Figure 3a), it is possible to observe three main peaks:

- the peak at 5203 cm^−1^ is related to the OH bond and is much higher for the Cristina cultivar than for Romina and Sibilla [32];
- the peak at 6866 cm^−1^ is assigned to C-H combinations and 8675 cm^−1^ to the 2nd overtone of C-H stretching, therefore it involves qualitative parameters such as the soluble solids content or the titratable acidity [32]. From the plot of spectra, it can be observed that the highest peak among the cultivar is reached by Romina, which is the cultivar with the highest soluble solids and the highest titratable acidity.

Two of the characteristic spectral bands detected on Figure 3a (6866 and 5203 cm^−1^) are also confirmed by the loading plot (Figure 3b).

### 3.3. PLS

Regression models for the prediction of the qualitative parameters of interest (soluble solids, firmness, acidity and color) have been developed. Prior to the computation of the PLS models, several pre-treatments of the spectral data were tested in order to minimize scatter and baseline effects [27]. For the soluble solids, firmness and color analyses, the dataset consisted of 800 observations (80 samples *x* 5 genotypes *x* 2 repetitions), while for the acidity the matrix was composed of 698 samples, because of some missing data. As for PCA, an outlier sample belonging to the Cristina cultivar has been deleted. The PLS regression models for the prediction of titratable acidity and color did not return good prediction performance and the models cannot be used for screening applications. The poor performance of the acid content prediction model may be due to the fact that the strawberries had all been harvested at the maturity stage, so they presented a low variation and a great uniformity of acidity values. The mean acidity value of the entire strawberry population analyzed was 0.64% of citric acid, with a standard deviation equal to 0.12 % of citric acid (data not shown), indicating the low variance of the examined samples and the related difficulties in developing a good regression model. The low standard deviation and the narrow range of acidity values make the PLS models not so good for predicting acidity-related parameters. These results are in line with the literature [13].

The regression models for the prediction of color were not satisfactory. PLS models were developed using both C*, the chromatic coordinates a*, b* and the brightness level L* as reference parameters (Y). None of these were suitable for the development of a satisfying prediction model. In any case, color is the only quality parameter already determined with a non-destructive technique, so the low performance of the prediction model has less impact compared to the acidity prediction model. In addition, a good model for color prediction is probably better obtained by using Vis-NIR spectrophotometer.

#### 3.3.1. Prediction of Soluble Solids Content

**Figure 4:**
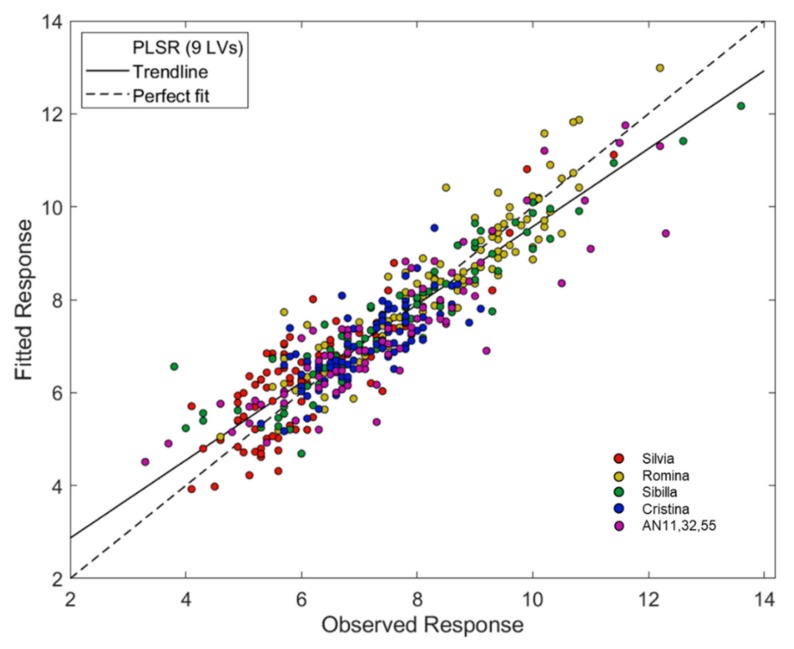
Regression plot of the Partial Least Squares Regression (PLS) model for the prediction of the soluble solids content obtained with Near Infrared (NIR) spectra pretreated with first derivatives (Savitzky–Golay filter, 21 smoothing points, 2nd polynomial order).

The PLS models for the prediction of the soluble solids content were very satisfactory. Several pre-treatment methods were tested before PLS computation (Table 2). The strongest PLS model was developed using a first derivative (Savitzky–Golay filter, 21 smoothing points, 2nd polynomial order) as pre-treatment. The model uses 9 LVs and returned *r*^2^ equal to 0.83, RMSECV 0.7 °Brix and bias = −0.002. RER = 14.71 and RPD = 2.41 demonstrated the good performance of the regression model. Figure 4 shows the regression plot of the PLS model for the prediction of the soluble solids content.

The best PLS model for the prediction of soluble solids content was also validated using an external test set. The spectra were pre-treated with first derivative (Savitzky–Golay method, 21 points window, second-order polynomial) and the model was developed using 9 LVs. It returned RMSECV = 0.7 °Brix, *r*^2^ = 0.83, bias = −0.019, RMSEP = 0.8 °Brix, *r*^2^ (pred) = 0.82. RPD = 1.04 and RER = 12.10 confirm the possibility to use the model for screening quality applications.

The results of this study are in line with those obtained by other authors. Amodio et al. [25] used an external test set to predict the soluble solid content of strawberry fruits with *r*^2^ = 0.85 and Root Mean Square Error of Prediction (RMSEP) 0.58%. Wlodarska et al. [23] also used an external test set for the prediction of strawberry (*r*^2^ (pred) = 0.93, RMSEP = 0.46%) and juices samples (*r*^2^ (pred) = 0.98, RMSEP = 0.25%) in the NIR range. The results of this study are even better than those of Sánchez et al. [13]. They used a LOCAL-PLS algorithm to predict the soluble solids content of nine strawberry cultivars and reported *r*^2^ = 0.69 and SEP (Standard Error of Prediction) = 0.88%. Shen et al. [24] studied the possibility to online predict the post-harvest quality of strawberry obtaining for soluble solids content *r*^2^ (pred) = 0.733, RMSEP = 0.699 °Brix and RPD 1.96.

**Figure 5:**
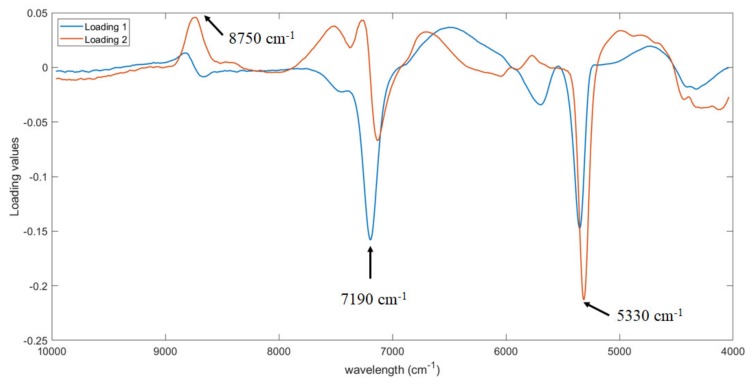
First two loadings of Partial Least Squares Regression (PLS) model for the prediction of soluble solid content.

The first two PLS loadings were analyzed to identify which wavelengths are more relevant for the prediction of the soluble solid content. As shown, three regions were observed in Figure 5, i.e., at around 8750, 7190 and 5330 cm^−1^. The same three regions were also detected by Amodio et al. [25]. In particular, the region from 5400 to 4720 cm^−1^ is assigned to OH, CH and CH_2_ deformation while the region from 8100 to 9100 cm^−1^ to C-H stretching [32]. Both bands are assigned to O-H and C-H bonds in water and sugar molecules [32,33].

#### 3.3.2. Prediction of Firmness

The PLS models for the prediction of firmness are less efficient than those of soluble solids, however they are still fair and therefore acceptable for screening applications. Several pre-treatments were evaluated (Table 3). The best one resulted, also in this case, in the first derivative (Savitzky–Golay filter, 21 smoothing points, 2nd polynomial order) which returned a model with *r*^2^ values equal to 0.54, RER = 10.36, RPD = 1.51, RMSECV = 0.11 g and bias = −0.0014. Based on the obtained indices, this model can be used to distinguish strawberries with high or low firmness values, helping to separate the product on the basis of the quality class. As for soluble solids content, the best PLS model for the prediction of firmness was also validated using an external test set. First derivative (Savitzky–Golay method, 21 points window, second-order polynomial) has been used as pre-treatment and the model was developed using five LVs. It returned RMSECV = 0.12 g, *r*^2^ = 0.45, bias = −0.0003, RMSEP = 0.15 g, *r*^2^ (pred) = 0.34. The indices pointed out a fair model that could be used for rough quality applications.

**Figure 6:**
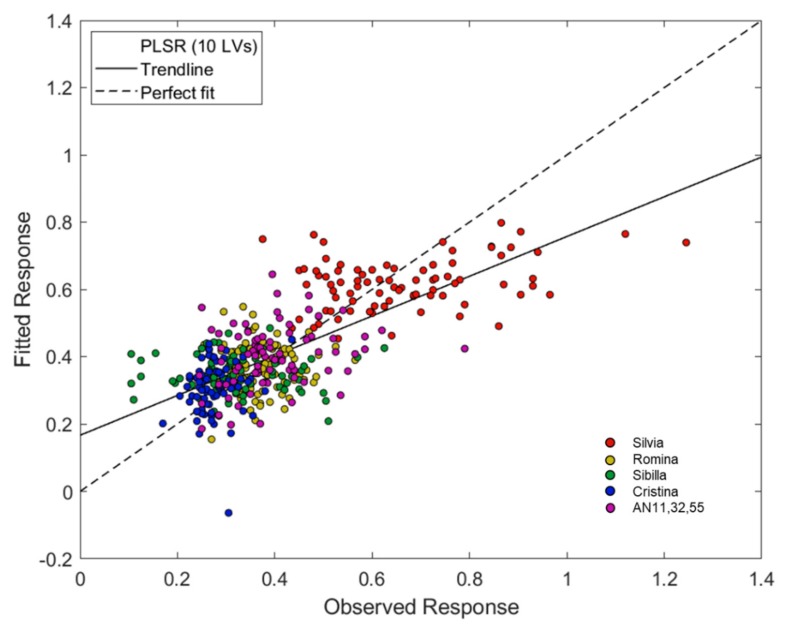
Regression plot of the Partial Least Squares Regression (PLS) model for the prediction of firmness obtained with Near Infrared (NIR) spectra pretreated with first derivatives (Savitzky–Golay filter. 21 smoothing points. 2nd polynomial order).

Figure 6 shows the regression plot of the PLS model for the prediction of firmness.

The results of this study are in line with those obtained by [13]. They predicted the firmness of nine strawberry cultivars using LOCAL-PLS algorithm obtaining *r*^2^ = 0.48 and SEP (Standard Error of Prediction) = 0.17 %.

**Figure 7:**
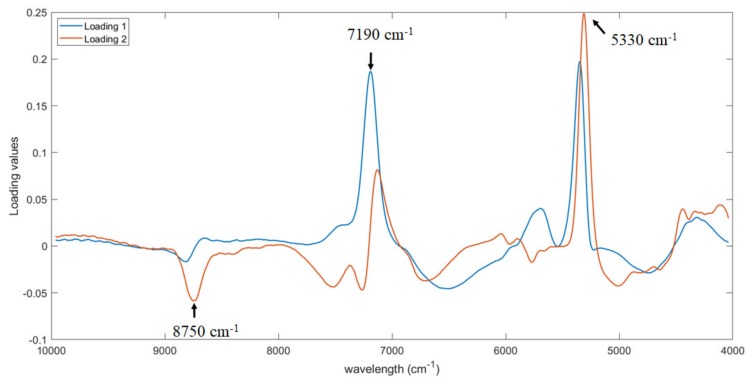
First two loadings of Partial Least Squares Regression (PLS) model for the prediction of firmness.

Furthermore, the first two PLS loadings were analyzed to identify the part of the signal more important for the prediction of firmness. The same three regions already detected for the prediction of soluble solid content were observed, i.e., at around 8750, 7190 and 5330 cm^−1^ with some minor shift (Figure 7). This is probably related to the fact that the more mature the fruit is, the higher the soluble solids content and the lower the firmness. As consequence, the same bands responsible for the prediction of soluble solid content are detected also for firmness prediction, but with an opposite trend (i.e., peak of maximum changes in a peak of minimum).

## 4. Conclusions

The results of this study showed that the PCA technique can be used to determine the spectral similarity of the fruits of different genotypes. In addition, this study demonstrated the possibility to use the FT-NIR technique coupled with PLS for the prediction of the qualitative parameters of strawberry fruits. In detail, the PLS model for the prediction of the soluble solids content resulted to be the most powerful model and it could be applied for any quality control application. A good PLS model was also developed for the prediction of firmness. The models obtained for the prediction of color and titratable acidity were not acceptable for screening control and further investigations should be carried on. Further efforts will be made to improve the predictive performance of the regression models, also investigating the feasibility of non-linear model applications.

The improvement and the use of this non-destructive technology in detecting the qualitative parameters of strawberry fruits has the potentiality to be very useful. In fact, NIR technology would be a multi-faceted instrument that can be used by researchers to select new cultivars with superior/target fruit nutritional quality and by all stakeholders in the fruit and vegetable supply chain (from the breeders, passing on to the most specialized agricultural entrepreneurs, up to buyers and large-scale retailers) for the fruit quality analysis on a large scale without fruit destruction. Besides the cultivars selection, this technique could lead to the separation of harvested fruits into “categories” (low acidity fruits, high soluble solids content fruits, firm fruits, soft fruits, and so on). Accordingly, consumers will be able to benefit from this supply chain by identifying the product that best meets their needs.

## Figures and Tables

**Table 1:** Results of fruit color (Brightness and Chroma), firmness, Soluble Solids Content (SS), and Titratable Acidity (TA). Data are expressed as means of 80 fruits ± standard deviation. Different superscript letters indicate significant differences at *p* < 0.05 (Tukey test).

Cultivars and Selections	Brightness (L*)	Chroma (C*)	Firmness (g)	SS (°Brix)	TA (% Citric Acid)
Romina	35.7 ± 2.1^c^	45.1 ± 4.0^d^	369 ± 58^b^	8.4 ± 1.5^a^	0.64 ± 0.14^b^
Sibilla	37.6 ± 2.2^b^	48.8 ± 2.7^b^	326 ± 102^c^	7.7 ± 1.9^ab^	0.55 ± 0.11^c^
Cristina	37.8 ± 1.9^b^	46.1 ± 3.1^c^	276 ± 40^d^	7.1 ± 0.9^b^	0.62 ± 0.07^b^
Silvia	36.4 ± 3.3^bc^	43.2 ± 5.1^d^	397 ± 95^b^	7.5 ± 1.8^ab^	0.77 ± 0.11^a^
AN11,32,55	39.4 ± 2.5^a^	51.5 ± 3.1^a^	661 ± 165^a^	6.0 ± 1.2^c^	0.65 ± 0.08^b^

**Table 2:** Summary of Partial Least Squares Regression (PLS) results for the prediction of soluble solid content (°Brix). The best PLS model is marked in bold (xder1 = first derivative with x number of smoothing points; xder2 = second derivative with x number of smoothing points; av = average; LVs = Latent Variables; RMSECV = Root Mean Square Error of Cross Validation; RER = Range Error Ratio; RPD = Ratio of Performance to Deviation).

Pre-Treatments	LVs	*r* ^2^	RMSECV (°Brix)	BIAS	RER	RPD
av	10	0.72	0.9	−0.0028	11.44	1.88
snv	8	0.66	0.98	0.0019	10.51	1.72
msc	8	0.67	0.98	−0.0025	10.51	1.72
9der1	9	0.8	0.75	−0.0079	13.73	2.25
13der1	9	0.83	0.71	−0.4049	14.51	2.38
**21der1**	**9**	**0.83**	**0.70**	−**0.0020**	**14.71**	**2.41**
9der2	1	0.58	1.64	0.0026	6.28	1.03
13der2	7	0.33	1.38	−0.0372	7.46	1.22
21der2	9	0.72	0.89	−0.0090	11.57	1.90

**Table 3:** Summary of Partial Least Squares Regression (PLS) results for the prediction of firmness (g). The best PLS model is marked in bold (xder1 = first derivative with x number of smoothing points; xder2 = second derivative with x number of smoothing points; av = average; LVs = Latent Variables; RMSECV = Root Mean Square Error of Cross Validation; RER = Range Error Ratio; RPD = Ratio of Performance to Deviation).

Pre-Treatments	LVs	*r* ^2^	RMSECV (g)	BIAS	RER	RPD
av	9	0.43	0.13	−0.0008	8.77	1.28
snv	10	0.41	0.13	−0.0013	8.77	1.28
msc	9	0.42	0.13	−0.0005	8.77	1.28
9der1	6	0.46	0.12	0.0003	9.50	1.38
13der1	5	0.42	0.13	−0.0003	8.77	1.28
**21der1**	**10**	**0.54**	**0.11**	−**0.0014**	**10.36**	**1.51**
9der2	2	0.30	0.14	0.0050	8.14	1.18
13der2	5	0.38	0.13	0.0039	8.77	1.28
21der2	5	0.43	0.13	0.0008	8.77	1.28

## References

[1] Di Vittori L., Mazzoni L., Battino M., Mezzetti B. (2018). Pre-harvest factors influencing the quality of berries. Sci. Hortic..

[2] Mezzetti B., Balducci F., Capocasa F., Zhong C.-F., Cappelletti R., Di Vittori L., Mazzoni L., Giampieri F., Battino M. (2016). Breeding Strawberry for Higher Phytochemicals Content and Claim It: Is It Possible?. Int. J. Fruit Sci..

[3] Capocasa F., Balducci F., Di Vittori L., Mazzoni L., Stewart D., Williams S., Hargreaves R., Bernardini D., Danesi L., Zhong C.-F. (2016). Romina and Cristina: Two New Strawberry Cultivars with High Sensorial and Nutritional Values. Int. J. Fruit Sci..

[4] Mazzoni L., Di Vittori L., Balducci F., Forbes-Hernández T., Giampieri F., Battino M., Mezzetti B., Capocasa F. (2020). Sensorial and nutritional quality of inter and intra—Specific strawberry genotypes selected in resilient conditions. Sci. Hortic..

[5] Darbellay C., Luisier J.-L., Villettaz J.-C., Azodanlou R. (2004). Changes in flavour and texture during the ripening of strawberries. Eur. Food Res. Technol..

[6] Clément A., Dorais M., Vernon M. (2008). Nondestructive Measurement of Fresh Tomato Lycopene Content and Other Physicochemical Characteristics Using Visible−NIR Spectroscopy. J. Agric. Food Chem..

[7] Aguayo E., Jansasithorn R., Kader A. (2006). Combined effects of 1-methylcyclopropene, calcium chloride dip, and/or atmospheric modification on quality changes in fresh-cut strawberries. Postharvest Boil. Technol..

[8] Mazzoni L., Álvarez-Suarez J.M., Giampieri F., Gasparrini M., Forbes-Hernández T., Mezzetti B. (2017). Evaluation of strawberry (Fragaria×ananassaDuch.) ‘Alba’ sensorial and nutritional quality, and its in vitro effects against human breast cancer cells viability. Acta Hortic..

[9] Butz P., Hofmann C., Tauscher B. (2006). Recent Developments in Noninvasive Techniques for Fresh Fruit and Vegetable Internal Quality Analysis. J. Food Sci..

[10] Giovenzana V., Beghi R., Civelli R., Guidetti R. (2015). Optical techniques for rapid quality monitoring along minimally processed fruit and vegetable chain. Trends Food Sci. Technol..

[11] Flores K., Sanchez M.-T., Perez-Marin D., Guerrero J.-E., Garrido-Varo A. (2009). Feasibility in NIRS instruments for predicting internal quality in intact tomato. J. Food Eng..

[12] Nicolaï B.M., Defraeye T., De Ketelaere B., Herremans E., Hertog M.L., Saeys W., Torricelli A., VandenDriessche T., Verboven P. (2014). Nondestructive Measurement of Fruit and Vegetable Quality. Annu. Rev. Food Sci. Technol..

[13] Sanchez M.-T., De La Haba M.J., Benítez-López M., Novales J.F., Garrido-Varo A., Perez-Marin D. (2012). Non-destructive characterization and quality control of intact strawberries based on NIR spectral data. J. Food Eng..

[14] Pissard A., Pierna J.A.F., Baeten V., Sinnaeve G., Lognay G., Mouteau A., Dupont P., Rondia A., Lateur M. (2012). Non-destructive measurement of vitamin C, total polyphenol and sugar content in apples using near-infrared spectroscopy. J. Sci. Food Agric..

[15] Porep J., Kammerer D.R., Carle R. (2015). On-line application of near infrared (NIR) spectroscopy in food production. Trends Food Sci. Technol..

[16] Sánchez N.N., Martínez-Marín A., Polvillo O., Fernández-Cabanás V., Carrizosa J., Urrutia B., Serradilla J. (2016). Near Infrared Spectroscopy (NIRS) for the determination of the milk fat fatty acid profile of goats. Food Chem..

[17] Toscano G., Rinnan Å., Pizzi A., Mancini M. (2017). The Use of Near-Infrared (NIR) Spectroscopy and Principal Component Analysis (PCA) To Discriminate Bark and Wood of the Most Common Species of the Pellet Sector. Energy Fuels.

[18] Mancini M., Rinnan Å., Pizzi A., Toscano G. (2018). Prediction of gross calorific value and ash content of woodchip samples by means of FT-NIR spectroscopy. Fuel Process. Technol..

[19] Pedersen T., Rantanen J., Naelapää K., Skibsted E. (2020). Near infrared analysis of pharmaceutical powders with empirical target distribution optimization (ETDO). J. Pharm. Biomed. Anal..

[20] Iyakwari S., Glass H., Rollinson G.K., Kowalczuk P.B. (2016). Application of near infrared sensors to preconcentration of hydrothermally-formed copper ore. Miner. Eng..

[21] Pasquini C. (2003). Near Infrared Spectroscopy: Fundamentals, practical aspects and analytical applications. J. Braz. Chem. Soc..

[22] Granato D., Putnik P., Kovačević D.B., Santos J.S., Calado V., Rocha R.S., Da Cruz A.G., Jarvis B., Rodionova O., Pomerantsev A.L. (2018). Trends in Chemometrics: Food Authentication, Microbiology, and Effects of Processing. Compr. Rev. Food Sci. Food Saf..

[23] Włodarska K., Szulc J., Khmelinskii I., Sikorska E. (2019). Non-destructive determination of strawberry fruit and juice quality parameters using ultraviolet, visible, and near-infrared spectroscopy. J. Sci. Food Agric..

[24] Shen F., Zhang B., Cao C., Jiang X. (2018). On-line discrimination of storage shelf-life and prediction of post-harvest quality for strawberry fruit by visible and near infrared spectroscopy. J. Food Process. Eng..

[25] Amodio M.L., Ceglie F.G., Chaudhry M.M.A., Piazzolla F., Colelli G. (2017). Potential of NIR spectroscopy for predicting internal quality and discriminating among strawberry fruits from different production systems. Postharvest Boil. Technol..

[26] Yazici A., Tiryaki G.Y., Ayvaz H. (2020). Determination of pesticide residual levels in strawberry (Fragaria) by near-infrared spectroscopy. J. Sci. Food Agric..

[27] Rinnan Å., Berg F.V.D., Engelsen S.B. (2009). Review of the most common pre-processing techniques for near-infrared spectra. TrAC Trends Anal. Chem..

[28] Wold S., Sjöström M., Eriksson L. (2001). PLS-regression: A basic tool of chemometrics. Chemom. Intell. Lab. Syst..

[29] Williams P., Norris K. (1987). Near-Infrared Technology in the Agricultural and Food Industries.

[30] Sørensen L.K. (2009). Application of reflectance near infrared spectroscopy for bread analyses. Food Chem..

[31] Fagan C., Everard C.D., McDonnell K. (2011). Prediction of moisture, calorific value, ash and carbon content of two dedicated bioenergy crops using near-infrared spectroscopy. Bioresour. Technol..

[32] Magwaza L.S., Opara U.O., Nieuwoudt H., Cronje P., Saeys W., Nicolaï B.M. (2011). NIR Spectroscopy Applications for Internal and External Quality Analysis of Citrus Fruit—A Review. Food Bioprocess Technol..

[33] Guthrie J.A., Walsh K.B., Reid D.J., Liebenberg C.J. (2005). Assessment of internal quality attributes of mandarin fruit. 1. NIR calibration model development. Aust. J. Agric. Res..

